# Triglyceride–glucose related index and its association with coronary heart disease risk in patients with metabolic dysfunction-associated steatotic liver disease: a retrospective analysis based on type 2 diabetes mellitus

**DOI:** 10.3389/fendo.2026.1839364

**Published:** 2026-06-16

**Authors:** Xuying Zhao, Yang Hong, Saiyan Bian, Hui Jiao, Xingjian Zhong, Wenkai Ni

**Affiliations:** 1 Department of Endocrinology and Metabolism, Affiliated Hospital of Nantong University, Nantong, Jiangsu, China; 2 Department of Gastroenterology, Affiliated Hospital of Nantong University, Nantong, Jiangsu, China; 3 Research Center of Clinical Medicine, Affiliated Hospital of Nantong University, Nantong, Jiangsu, China; 4 Department of Endocrinology and Metabolism, The Second People’s Hospital of, Kunshan, Jiangsu, China; 5 Medical College of Nantong University, Nantong, Jiangsu, China

**Keywords:** coronary heart disease (CHD), metabolic dysfunction-associated steatotic liver disease (MASLD), restricted cubic spline (RCS), triglyceride–glucose (TyG), type 2 diabetes mellitus (T2DM)

## Abstract

**Background:**

The triglyceride–glucose (TyG) related index, as an emerging indicator of insulin resistance, has been shown to be closely associated with the clinical status (incidence of cardiovascular disease, all-cause mortality, etc.) in patients with MASLD. However, differences in this association among patients with or without type 2 diabetes mellitus (T2DM) have not yet been confirmed. This study aims to address this gap.

**Methods:**

This cross-sectional study included 812 patients with MASLD who visited our hospital from January 2021 to January 2025. Patients were divided into two groups based on the presence or absence of coronary heart disease (CHD). Univariate analysis was performed to compare baseline characteristics between groups. Univariate and multivariate logistic analyses were conducted to assess the associations between various biochemical indices, including TyG, and the risk of CHD. An interaction term between TyG and T2DM was constructed to evaluate the effect modification of diabetes status on this association. The performance of multivariate logistic regression models with and without the inclusion of T2DM was compared. Dose–response relationships between TyG and CHD risk under different T2DM statuses were explored, and receiver operating characteristic (ROC) curves were plotted.

**Results:**

TyG showed a significant positive correlation with the risk of CHD (OR = 2.035). T2DM was also significantly positively associated with CHD risk (OR = 2.107). Several biochemical indices, including LDL, TG, ALT, AST, GGT, Scr, and HbA1c, were identified as associated factors for CHD (OR > 1), while HDL and eGFR were protective factors (OR < 1). Interaction analysis indicated that the association between TyG and CHD was significantly stronger in patients with T2DM, with an OR of 3.34, which was substantially higher than the OR of 1.89 observed in patients without T2DM. Restricted cubic spline (RCS) analysis demonstrated a linear relationship between TyG and CHD risk in both patients with and without T2DM. The ROC-derived cut-off value for TyG was 7.992 in patients without T2DM and 9.005 in patients with T2DM; beyond these cut-off values, the risk of CHD increased significantly.

**Conclusion:**

The TyG index shows a significant positive correlation with the risk of CHD in patients with MASLD, and this correlation is modified by T2DM.

## 1 Introduction

Metabolic dysfunction-associated steatotic liver disease (MASLD) is a recently proposed concept, previously known as non-alcoholic fatty liver disease (NAFLD). It is a common liver disease affecting 38% of adults globally (1) ^(^2^),^. This condition results from excessive fat accumulation in hepatocytes, not caused by alcohol or other obvious liver injuries, and its pathogenesis is primarily driven by insulin resistance and metabolic abnormalities. The disease spectrum of MASLD includes simple steatosis, metabolic dysfunction-associated steatohepatitis (MASH) (3), liver fibrosis (4), cirrhosis, and hepatocellular carcinoma (HCC) (5). MASLD often coexists with chronic conditions such as obesity (6), insulin resistance (7) ^(^8^),^, and type 2 diabetes mellitus (T2DM), and is considered an important manifestation of systemic metabolic disorders. In recent years, an increasing number of studies have shown that MASLD is closely associated with various cardiovascular diseases (9), particularly coronary heart disease (CHD). Mohammad et al. elucidated that MASLD promotes the occurrence and progression of atherosclerosis through mechanisms including insulin resistance, dyslipidemia, inflammation, and endothelial dysfunction, thereby significantly increasing the risk of CHD (10).

Triglyceride–glucose (TyG) is a simple index calculated from fasting blood glucose and triglycerides and has gradually been recognized as a reliable surrogate marker of insulin resistance. The core value of TyG lies in its ability to reflect insulin resistance while integrating information on glucose and lipid metabolism. Compared with traditional insulin resistance assessment methods, TyG is simpler to obtain and more cost-effective. TyG holds significant clinical importance in patients with MASLD. Studies by Yusha et al. have demonstrated that TyG has substantial predictive value for all-cause mortality and cardiovascular risk in patients with MASLD (11). Wenyuan et al. further validated the positive correlation between TyG and CHD risk in patients with MASLD (12).

However, patients with T2DM typically present with hyperinsulinemia, systemic inflammation, and more severe metabolic disturbances (13). Most existing studies have not differentiated patients with MASLD based on T2DM status, and the relationship between TyG and CHD risk has not been systematically evaluated under this condition. This study aims to address this gap by stratifying according to T2DM status to comprehensively evaluating the diagnostic accuracy of TyG in predicting CHD risk in patients with MASLD and to provide reference cut-off values.

## 2 Materials and methods

### 2.1 Study subjects

This cross-sectional study ultimately included 812 patients with MASLD who visited our hospital from January 2021 to January 2025. Patients were divided into two groups based on the presence or absence of CHD. Inclusion criteria were as follows: 1) age ≥ 18 years; 2) met the diagnostic criteria for MASLD: first, hepatic steatosis was assessed using standard enhanced liver ultrasound; further evaluation was conducted for the presence of cardiometabolic associated factors (overweight or obesity, abnormal glucose metabolism or type 2 diabetes mellitus, plasma triglycerides ≥ 1.7 mmol/L, high-density lipoprotein-cholesterol ≤ 1.0 mmol/L, blood pressure ≥ 130/85 mmHg); finally, alcohol consumption was excluded (alcohol intake ≤ 20 g/day for females and ≤ 30 g/day for males); all the above conditions were required to be met (14); 3) complete clinical information. Exclusion criteria were as follows: 1) other types of heart disease (e.g., rheumatic heart disease, valvular heart disease, severe heart failure, etc.); 2) previous use of statins or triglyceride-lowering medications; 3) presence of other severe systemic diseases including advanced cancer, end-stage renal disease, autoimmune diseases, etc.; 4) missing key data including inability to determine T2DM status or CHD diagnosis.

The diagnostic criteria for coronary heart disease were stenosis ≥ 50% in any major coronary artery or other important branches as shown by coronary angiography.

### 2.2 Data collection

Demographic information including age, sex, body mass index (BMI), smoking status, alcohol consumption, family history of liver disease, hypertension, and type 2 diabetes mellitus (T2DM) was collected from electronic medical records. Fasting venous blood samples (fasting for 8–12 hours) were collected from all study subjects at baseline for analysis. The measured indicators were total cholesterol (TC), high-density lipoprotein cholesterol (HDL-C), low-density lipoprotein cholesterol (LDL-C), and triglycerides (TG); alanine aminotransferase (ALT), aspartate aminotransferase (AST), gamma-glutamyl transferase (GGT), glycated hemoglobin (HbA1c), and serum creatinine (SCr). The estimated glomerular filtration rate (eGFR), TyG, and TyG-BMI index were calculated according to the following formulas:


$$TyG=\text{ln}(\frac{TG\times FPG}{2})$$

TyG-BMI=TyG×BMI

Prior to calculating the TyG index, unit conversion of the original biochemical parameters was performed (mmol/L converted to mg/dL).

### 2.3 Statistical analysis

All statistical analyses and graphing in this study were performed using R software (version 4.4.1). Continuous data were expressed as median (minimum–maximum), and comparisons were made using the t-test or Mann–Whitney U test. Categorical variables were expressed as number of cases (percentage), and intergroup comparisons were performed using the χ² test or Fisher’s exact test. To control for the risk of false positives due to multiple comparisons, the false discovery rate (FDR) was used for correction. All statistical tests were two-sided, and a P-value < 0.05 was considered statistically significant. Univariate and multivariate logistic regression analyses were used to screen for potential factors associated with CHD occurrence, in order to evaluate the independent associations between TyG and other biochemical indicators and CHD risk. Interaction terms between TyG and other significant factors were constructed in the models to assess interactions. The receiver operating characteristic (ROC) curve was used to evaluate the performance of the multivariable logistic regression models without T2DM (Model 1) and with T2DM (Model 2). Restricted cubic spline (RCS) analysis was used to examine the dose–response relationship between TyG and CHD risk and to explore potential nonlinear associations. ROC curves were also plotted to evaluate the discriminative ability of TyG for CHD. Regarding the handling of missing data, when missing data in a sample accounted for less than 5%, median imputation was used; when missing data accounted for ≥5% and <20%, multiple imputation was used; when the proportion of missing data was ≥20%, the sample was directly excluded. We calculated the required sample size under the condition of a medium effect size (d = 0.5) with a statistical power of 80%. For continuous data, the theoretical requirement was at least 64 samples per group; for categorical data, the theoretical requirement was at least 63 samples per group. The actual sample size in this study exceeded the theoretical minimum in all cases, indicating that this study had adequate statistical power.

## 3 Results

### 3.1 Baseline characteristics differences between CHD and non-CHD patients

BMI was significantly higher in the CHD group than in the non-CHD group (P = 0.00021, FDR = 0.00084). The proportions of family history of liver disease (P = 0.0046, FDR = 0.0092), hypertension (P = 0.0021, FDR = 0.0055), and type 2 diabetes mellitus (P < 0.0001, FDR = 0.00015) were also significantly higher in the CHD group compared with the non-CHD group (**Table 1**).

**Table 1:** Differences in baseline characteristics between CHD and non-CHD patients.

Characteristics	TOTAL (n=812)	Non-CHD (n= 587)	CHD (n= 225)	P-value	FDR
Age	46 (37-55)	46 (37-55)	46 (37-55)	0.282	0.451
Gender (Male)	450 (55.42%)	329 (56.05%)	121 (53.78%)	0.615	0.702
BMI	24.9 (19.1-29.7)	24.8 (19.1-29.1)	25.2 (19.9-29.7)	< 0.001	0.001
Smoking	257 (31.65%)	184 (31.35%)	73 (32.44%)	0.828	0.828
Drinking	318 (39.16%)	224 (38.16%)	94 (41.78%)	0.387	0.516
Family history of liver disease	137 (16.87%)	85 (14.48%)	52 (23.11%)	0.005	0.009
Hypertension	375 (46.18%)	251 (42.76%)	124 (55.11%)	0.002	0.006
Type 2 Diabetes Mellitus	221 (27.22%)	135 (23%)	86 (38.22%)	< 0.001	< 0.001

### 3.2 Biochemical indicator differences between CHD and non-CHD patients

TC, LDL, TG, AST, ALT, GGT, SCr, HbA1c, TyG, and TyG-BMI were significantly higher in the CHD group than in the non-CHD group, while HDL and eGFR were significantly lower in the CHD group compared with the non-CHD group (**Table 2**).

**Table 2:** Differences in biochemical parameters between CHD and non-CHD groups.

Biochemical parameters	TOTAL (n=812)	Non-CHD (n= 587)	CHD (n= 225)	P-value	FDR
Total Cholesterol (TC) (mmol/L)	4.8 (4.1-5.9)	4.8 (4.1-5.9)	4.9 (4.3-5.8)	0.039	0.042
High-Density Lipoprotein Cholesterol (HDL-C) (mmol/L)	1.3 (1.0-1.6)	1.3 (1.0-1.6)	1.2 (1.0-1.6)	0.001	0.004
Low-Density Lipoprotein Cholesterol (LDL-C) (mmol/L)	3.3 (2.5-4.1)	3.2 (2.5-4.1)	3.3 (2.8-4.1)	< 0.001	0.001
Serum Triglycerides (TG) (mmol/L)	1.6 (0.6-2.9)	1.6 (0.6-2.6)	1.7 (0.7-2.9)	0.006	0.012
Alanine Aminotransferase (ALT) (U/L)	37.6 (22.6-55.2)	37.1 (22.6-55.2)	38.8 (27.4-53.0)	0.003	0.006
Aspartate Aminotransferase (AST) (U/L)	33.9 (21.1-49.9)	33.6 (21.1-49.9)	34.4 (25.4-49.1)	0.009	0.012
amma-Glutamyl Transferase (GGT) (U/L)	35.1 (23.2-51.0)	34.6 (23.2-51.0)	36.1 (26.2-50.4)	0.008	0.012
Serum Creatinine (SCr) (μmol/L)	61.4 (38.3-88.5)	60.8 (40.8-82.7)	63.0 (38.3-88.5)	0.001	0.004
Estimated Glomerular Filtration Rate (eGFR) (mL/min/1.73 m²)	106.1 (83.0-129.3)	106.4 (83.0-129.3)	105.0 (87.4-126.3)	0.043	0.043
Glycated Hemoglobin (HbA1c)(%)	5.2 (4.0-7.5)	5.2 (4.0-7.5)	5.3 (4.1-7.4)	0.008	0.012
Triglyceride–glucose (TyG)	7.9 (7.2-9.3)	7.8 (7.2-9.3)	7.9 (7.2-9.0)	0.016	0.019
TyG-BMI	230.7 (199.3-276.2)	229.4 (202.3-276.2)	234.7 (199.3-269.3)	0.001	0.004

### 3.3 Univariate and multivariate logistic regression analysis of CHD associated factors

With the exception of TC, all significant factors from sections 3.1 and 3.2 remained significant in both univariate and multivariate logistic regression analyses, including BMI, family history of liver disease, hypertension, type 2 diabetes mellitus (T2DM), HDL, LDL, TG, ALT, AST, GGT, serum creatinine (Scr), eGFR, HbA1c, TyG, and TyG-BMI. Notably, HDL and eGFR were protective factors (OR < 1), while the remaining factors were associated factors (OR > 1) (**Table 3**).

**Table 3:** Univariate and multivariable logistic regression analysis of risk factors for CHD.

Variables	Univariate				Multivariable (model 1)				
	OR	CI-lower	CI-upper	P-value	Coef	P-value	OR	CI-lower	CI-upper
BMI	1.192	1.091	1.305	0.000	0.180	0.000	1.197	1.083	1.323
Family history of liver disease	1.775	1.202	2.604	0.004	0.661	0.003	1.937	1.253	2.994
Hypertension	1.643	1.207	2.242	0.002	0.443	0.012	1.557	1.104	2.196
T2DM	2.072	1.487	2.882	0.000	0.745	0.000	2.107	1.461	3.039
TC	1.582	0.940	2.655	0.083	–	–	–	–	–
HDL	0.056	0.011	0.287	0.001	-3.117	0.001	0.044	0.007	0.270
LDL	3.174	1.778	5.708	0.000	1.205	0.000	3.337	1.746	6.378
TG	1.963	1.218	3.179	0.006	0.622	0.023	1.863	1.092	3.181
ALT	1.045	1.017	1.074	0.002	0.040	0.010	1.041	1.010	1.074
AST	1.044	1.008	1.081	0.016	0.048	0.015	1.049	1.009	1.091
GGT	1.048	1.011	1.086	0.011	0.052	0.010	1.054	1.013	1.097
Scr	1.032	1.012	1.053	0.002	0.030	0.008	1.030	1.008	1.053
eGFR	0.976	0.954	0.998	0.035	-0.025	0.000	0.976	0.963	0.988
HbA1c	1.438	1.101	1.879	0.008	0.294	0.051	1.342	0.999	1.802
TyG	1.672	1.048	2.666	0.031	0.710	0.008	2.035	1.204	3.439
TyG-BMI	1.018	1.005	1.031	0.006	0.021	0.002	1.022	1.008	1.036

BMI, Body Mass Index; T2DM, Type 2 Diabetes Mellitus; TC, Total Cholesterol; HDL, High-Density Lipoprotein Cholesterol; LDL, Low-Density Lipoprotein Cholesterol; TG, Triglycerides; ALT, Alanine Aminotransferase; AST, Aspartate Aminotransferase; GGT, Gamma-Glutamyl Transferase; Scr, Serum Creatinine; eGFR, Estimated Glomerular Filtration Rate; HbA1c, Glycated Hemoglobin; TyG, Triglyceride-Glucose Index; TyG-BMI, TyG multiplied by BMI.

### 3.4 Interaction analysis of TyG

**Figure 1:**
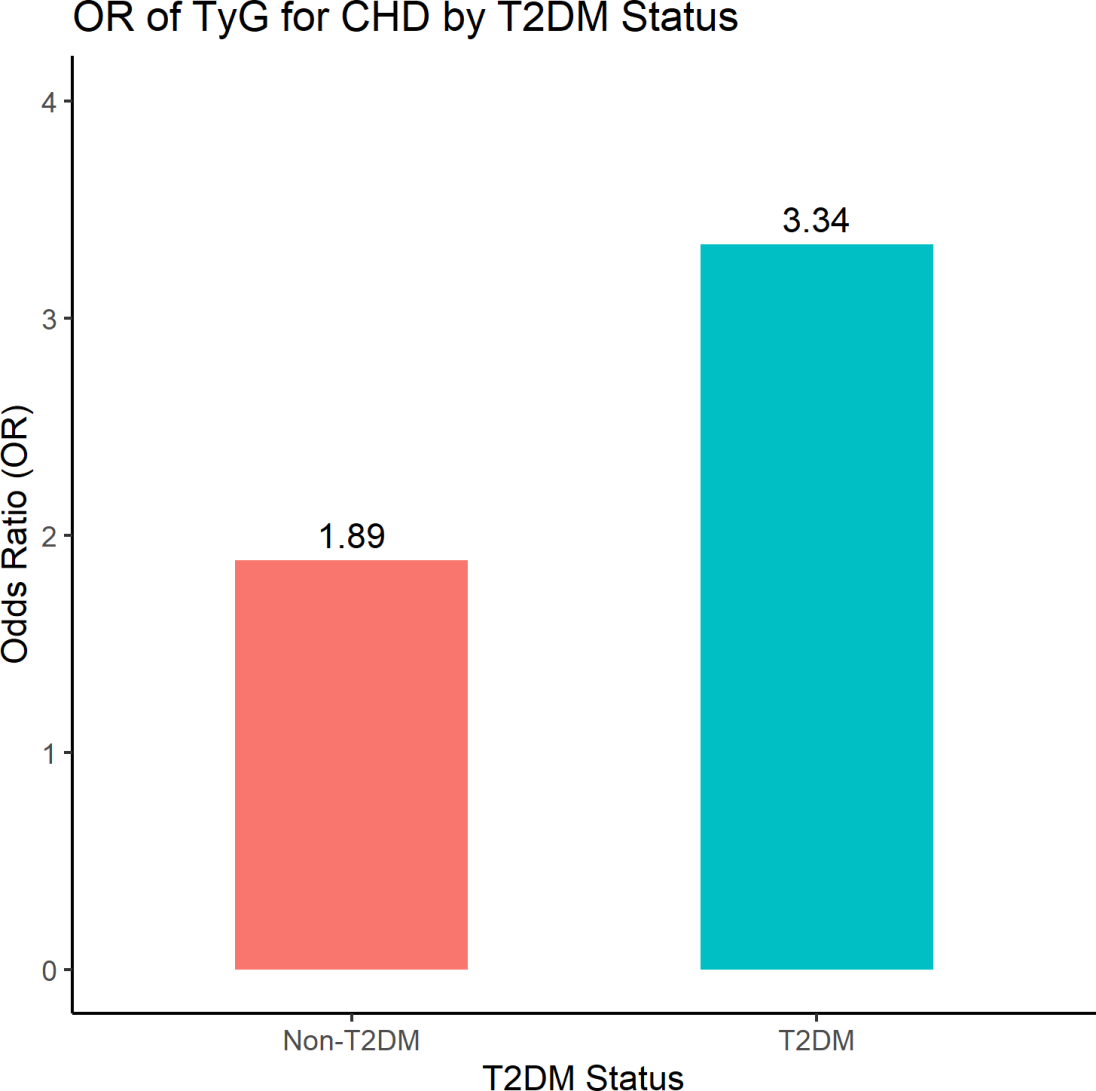
Effect of TyG (OR) under different T2DM statuses.

The results indicated that in the interaction analysis between TyG and T2DM, both TyG (OR 1.887, 95% CI 1.071–3.325, P = 0.028) and T2DM (OR 5.675, 95% CI 1.204–26.745, P = 0.028) were significant, and the TyG × T2DM interaction term was also significant (OR 1.772, 95% CI 1.303–2.409, P < 0.001). The OR of the interaction term was greater than 1, indicating that the association between TyG and CHD was stronger in patients with T2DM. Based on the logistic regression formula, we calculated the OR values for TyG in patients with and without T2DM, respectively (**Figure 1**). In the interaction analyses of other indicators with TyG, none of the interaction terms showed statistical significance (P > 0.05) (**Table 4**).

**Table 4:** Interaction analysis of TyG.

Interaction Term	OR (95% CI)	p-value	Coef
TyG	1.926 (1.132–3.277)	0.016	0.655
TyG * Family history of liver disease	0.487 (0.158–1.508)	0.212	-0.719
TyG	1.887 (1.071–3.325)	0.028	0.635
TyG * T2DM	1.772 (1.303–2.409)	0.000	0.572
TyG	1.987 (0.178–22.190)	0.577	0.687
TyG * HDL	2.285 (0.325–15.826)	0.403	0.826
TyG	1.341 (0.034–52.875)	0.876	0.293
TyG * LDL	1.063 (0.175–6.463)	0.947	0.061
TyG	2.541 (0.410–15.753)	0.316	0.933
TyG * ALT	1.034 (0.949–1.126)	0.446	0.033
TyG	1.721 (0.126–23.507)	0.684	0.543
TyG * Scr	0.981 (0.923–1.042)	0.534	-0.019
TyG	3.101 (0.739–13.011)	0.122	1.132
TyG * eGFR	0.973 (0.940–1.008)	0.132	-0.027

TyG, Triglyceride-Glucose Index; T2DM, Type 2 Diabetes Mellitus; HDL, High-Density Lipoprotein Cholesterol; LDL, Low-Density Lipoprotein Cholesterol; ALT, Alanine Aminotransferase; Scr, Serum Creatinine; eGFR, Estimated Glomerular Filtration Rate.

“*” indicates an interaction term.

### 3.5 ROC curve analysis of multivariate logistic regression models with and without T2DM

**Figure 2:**
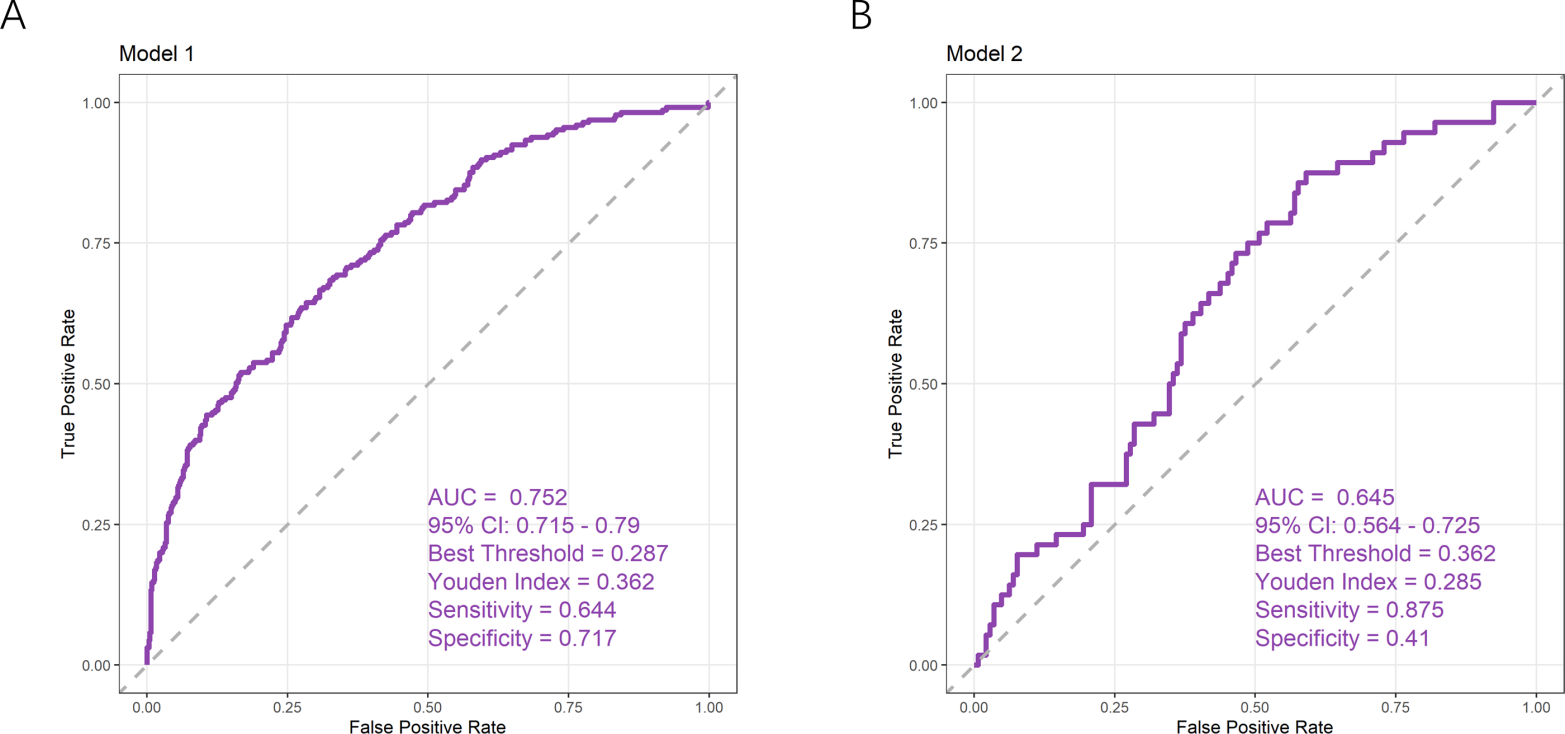
**(A)** ROC curve of the multivariate logistic regression model including T2DM; **(B)** ROC curve of the multivariate logistic regression model excluding T2DM.

When T2DM was included as a variable (Model 1), the AUC value of the multivariate logistic regression model was 0.752. When T2DM was removed from the model (Model 2), the AUC value of the multivariate logistic regression model was only 0.645. The DeLong test indicated that the P-value for the comparison of these two ROC curves was 0.018, suggesting that the discriminative ability of the model was significantly improved after including T2DM, highlighting its important value in risk assessment (**Figures 2A, B**).

### 3.6 RCS analysis under different T2DM statuses

**Figure 3:**
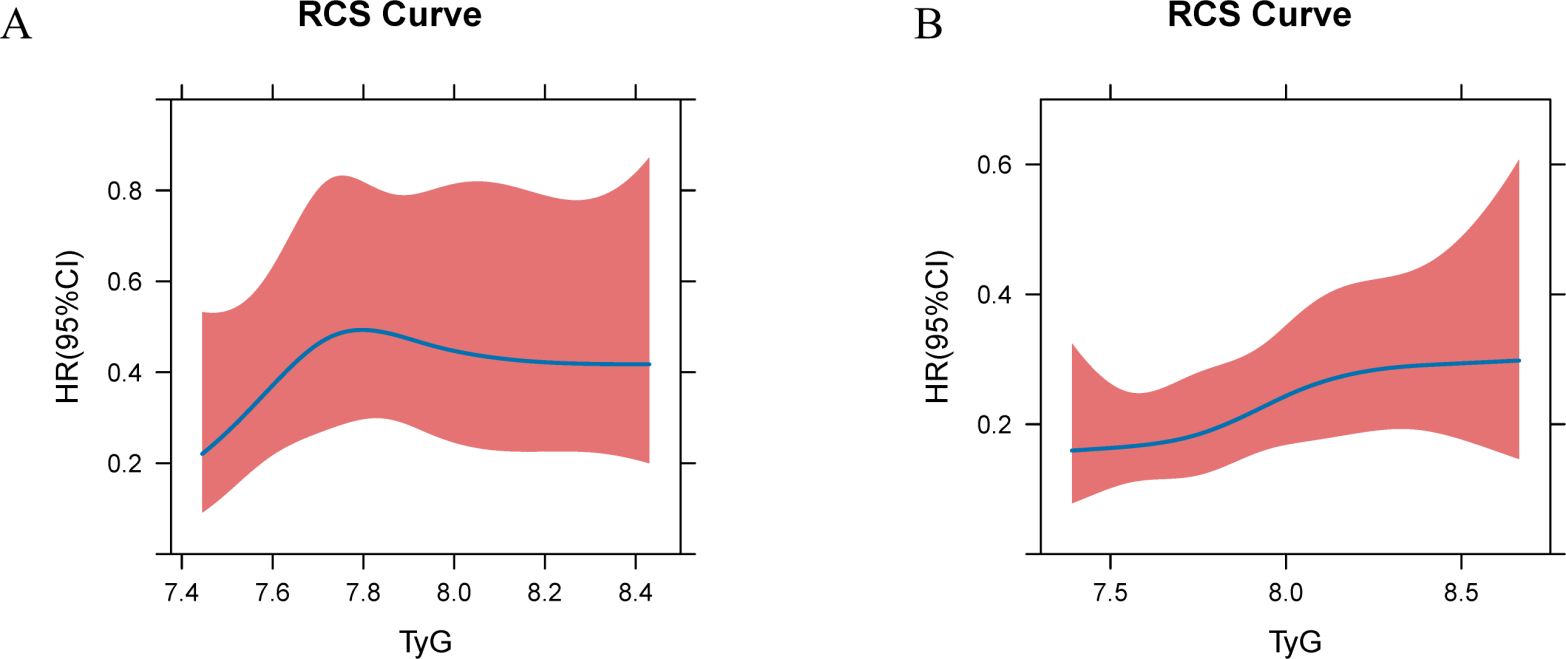
**(A)** RCS curve under T2DM status; **(B)** RCS curve under non-T2DM status.

In patients with T2DM, the risk of CHD gradually increased with increasing TyG levels (P for overall = 0.0048), while nonlinear analysis indicated a non-significant nonlinear relationship between TyG and CHD risk, suggesting that the association between the two is linear (P for nonlinear = 0.3495). The same pattern was observed in patients without T2DM (P for overall = 0.0016, P for nonlinear = 0.7930) (**Figures 3A, B**).

### 3.7 ROC curves of TyG for discriminating CHD under different T2DM statuses

**Figure 4:**
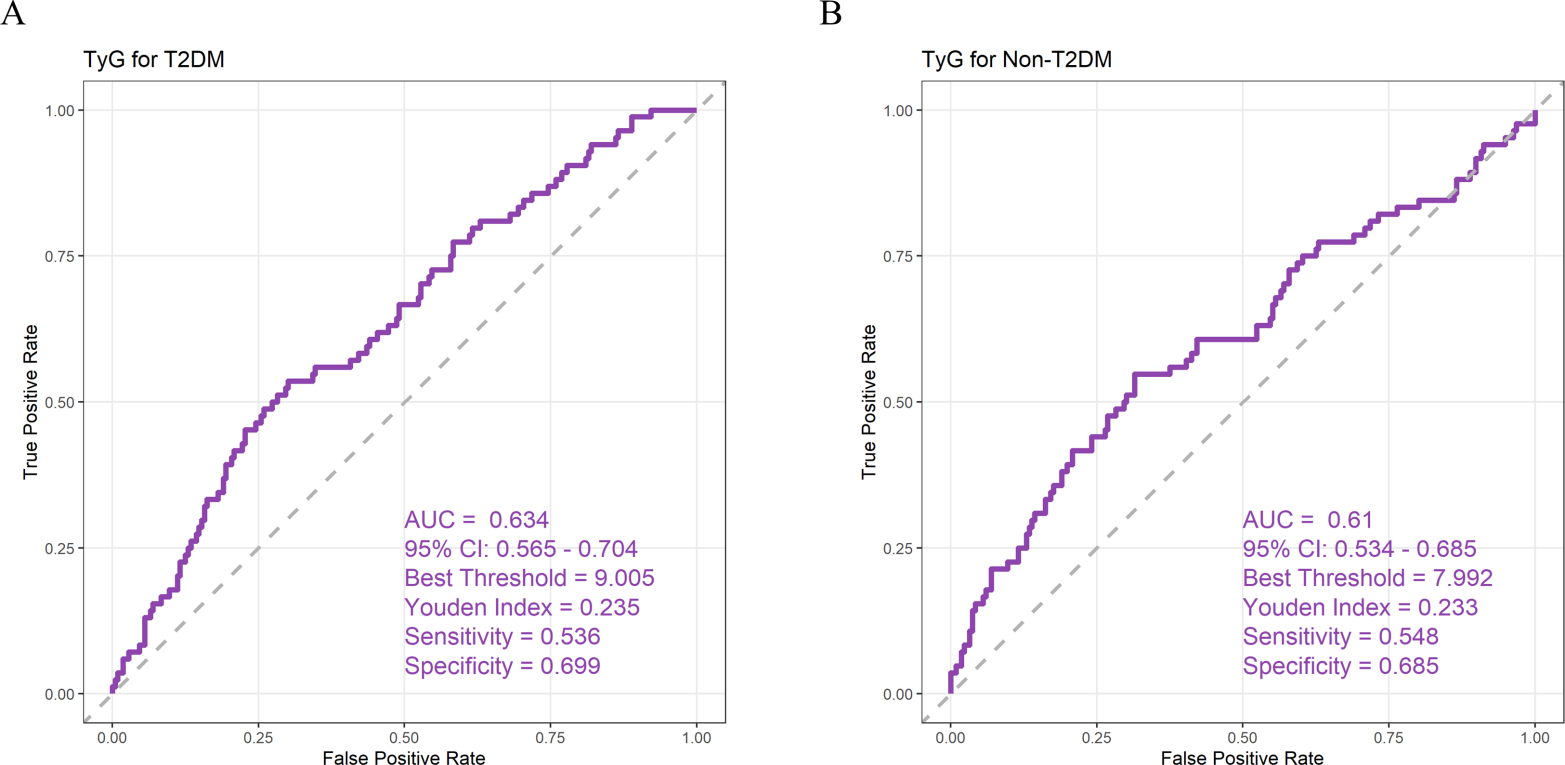
**(A)** ROC curve of TyG for discriminating CHD in patients with T2DM; **(B)** ROC curve of TyG for discriminating CHD in patients without T2DM.

In patients with T2DM, the TyG index showed moderate discriminative ability for CHD, with an AUC of 0.634 (95% CI: 0.565–0.704) and an optimal cutoff value of 9.005. In patients without T2DM, the discriminative ability of the TyG index for CHD was lower than that in patients with T2DM, with an AUC of 0.610 (95% CI: 0.534–0.685) and an optimal cutoff value of 7.992 (**Figures 4A, B**).

## 4 Discussion

This study found that multiple biochemical indicators differed significantly between MASLD patients with CHD and those without CHD, including elevated blood lipids, elevated liver function indicators, elevated glycemic control indicators, and decreased renal function indicators. This suggests that the occurrence of CHD in MASLD patients is closely associated with multiple metabolic disturbances, reflecting the cumulative role of insulin resistance, lipid metabolism abnormalities, and organ function impairment in cardiovascular risk.

We observed that TyG, as an important indicator reflecting insulin resistance (15), was more strongly associated with CHD in patients with T2DM, with its effect size approximately twice that in patients without T2DM. This may be because in patients with MASLD and coexisting T2DM, patients not only have hepatic steatosis but also significant hyperglycemia. In this context, elevated TyG represents not merely an increase in two numerical values, but rather indicates extreme oxidative stress. High glucose and high free fatty acids jointly activate pathways such as protein kinase C (PKC) and nuclear factor κB (NF-κB), leading to severe impairment of endothelial cell function (16) ^(^17^),^. This synergistic effect of glucotoxicity and lipotoxicity on vascular wall damage is far greater than that of isolated lipid metabolism abnormalities, as MASLD patients without T2DM often predominantly exhibit lipotoxicity alone. In patients with MASLD, the liver itself overproduces very low-density lipoprotein (VLDL), which promotes atherosclerosis (18). When T2DM is also present, hyperinsulinemia directly promotes vascular smooth muscle proliferation, stimulates endothelin-1 (ET-1) secretion resulting in vasoconstriction, while simultaneously inhibiting nitric oxide (NO) production and suppressing vasodilation (19). These factors collectively amplify the damaging effect of TyG on vasculature in T2DM patients. In MASLD patients without T2DM, inflammation is primarily localized to the liver, whereas in MASLD patients with T2DM, the inflammatory response induced by hyperglycemia itself further extends the inflammatory response systemically (20). As TyG is an indicator associated with inflammatory markers, its indicative value for CHD risk is therefore significantly amplified in patients with T2DM.

This study also has certain clinical implications. These findings suggest that when assessing TyG in relation to CHD risk in patients with MASLD, attention should be paid to whether the patient has T2DM. The ROC curve results indicate that for patients without T2DM, when the TyG level exceeds 7.992, the risk of CHD begins to increase significantly, suggesting that this cut-off value may serve as a reference indicator for early identification of high-risk individuals among the non-T2DM population. In patients with T2DM, when the TyG level exceeds 9.005, the risk of CHD begins to increase significantly; this cut-off value can similarly serve as a reference indicator for early identification of individuals at high CHD risk among patients with MASLD and coexisting T2DM. Clinicians can use these two cut-off values to assist in evaluating cardiovascular risk and to develop more precise protective measures based on the patient’s T2DM status, such as improving dietary habits to optimize glycemic and lipid control, and adopting healthier lifestyles.

The diagnostic criteria used in this study differ from the NAFLD diagnostic criteria used in previous studies. For example, Jianqi et al. also investigated the association between TyG and CHD risk (21), but their diagnostic criteria were based on the old NAFLD criteria, which relied on exclusionary diagnosis (excluding alcohol, viruses, medications, etc.) without emphasizing metabolic abnormalities as the core. In contrast, this study used the new MASLD criteria, which require the presence of at least one cardiometabolic associated factor, making the results more targeted and allowing the findings to directly align with the latest international consensus and clinical guidelines, thereby enhancing generalizability and impact.

This study also has certain limitations. First, as a retrospective study, there is some degree of selection bias. Due to the lack of longitudinal follow-up data, we were unable to assess the temporal relationship between the TyG index and the occurrence of CHD. The present results only reflect a cross-sectional association and cannot be used to infer causality. Because the diagnosis of CHD requires coronary angiography, some asymptomatic or subclinical CHD patients may not be identified and may be classified into the non-CHD group, leading to misclassification bias. Our data collection was also not comprehensive, and other potential confounding factors, such as medication use, remain. Future multicenter prospective cohort studies with longitudinal follow-up that incorporate confounding factors can be conducted to further validate the causal association between TyG and CHD. Hepatic ultrasonography has limited sensitivity for mild hepatic steatosis, which may lead to underestimation of mild fatty liver. This study also did not further analyze the degree of fibrosis in patients with MASLD. Future studies could further investigate the association between TyG and the risk of CHD by incorporating fibrosis staging.

## 5 Conclusion

This study demonstrates a positive correlation between TyG and CHD risk, and this correlation is stronger in patients with T2DM. The cut-off value for TyG is 7.992 in patients without T2DM and 9.005 in patients with T2DM. For patients with TyG levels exceeding the corresponding cut-off values, attention should be paid to their potential cardiovascular risk, and a comprehensive assessment should be conducted in conjunction with clinical and other risk factors.

## Data Availability

The raw data supporting the conclusions of this article will be made available by the authors, without undue reservation.
